# Mitigation of building-related polychlorinated biphenyls in indoor air of a school

**DOI:** 10.1186/1476-069X-11-24

**Published:** 2012-04-10

**Authors:** David L MacIntosh, Taeko Minegishi, Matthew A Fragala, Joseph G Allen, Kevin M Coghlan, James H Stewart, John F McCarthy

**Affiliations:** 1 Environmental Health & Engineering, Inc, 117 Fourth Avenue, Needham, MA 02494-2705, USA

**Keywords:** Remediation, Abatement, Flux, Risk management

## Abstract

**Background:**

Sealants and other building materials sold in the U.S. from 1958 - 1971 were commonly manufactured with polychlorinated biphenyls (PCBs) at percent quantities by weight. Volatilization of PCBs from construction materials has been reported to produce PCB levels in indoor air that exceed health protective guideline values. The discovery of PCBs in indoor air of schools can produce numerous complications including disruption of normal operations and potential risks to health. Understanding the dynamics of building-related PCBs in indoor air is needed to identify effective strategies for managing potential exposures and risks. This paper reports on the efficacy of selected engineering controls implemented to mitigate concentrations of PCBs in indoor air.

**Methods:**

Three interventions (ventilation, contact encapsulation, and physical barriers) were evaluated in an elementary school with PCB-containing caulk and elevated PCB concentrations in indoor air. Fluorescent light ballasts did not contain PCBs. Following implementation of the final intervention, measurements obtained over 14 months were used to assess the efficacy of the mitigation methods over time as well as temporal variability of PCBs in indoor air.

**Results:**

Controlling for air exchange rates and temperature, the interventions produced statistically significant (p < 0.05) reductions in concentrations of PCBs in indoor air of the school. The mitigation measures remained effective over the course of the entire follow-up period. After all interventions were implemented, PCB levels in indoor air were associated with indoor temperature. In a "broken-stick" regression model with a node at 20°C, temperature explained 79% of the variability of indoor PCB concentrations over time (p < 0.001).

**Conclusions:**

Increasing outdoor air ventilation, encapsulating caulk, and constructing a physical barrier over the encapsulated material were shown to be effective at reducing exposure concentrations of PCBs in indoor air of a school and also preventing direct contact with PCB caulk. In-place management methods such as these avoid the disruption and higher costs of demolition, disposal and reconstruction required when PCB-containing building materials are removed from a school. Because of the influence of temperature on indoor air PCB levels, risk assessment results based on short-term measurements, e.g., a single day or season, may be erroneous and could lead to sub-optimal allocation of resources.

## Introduction

PCBs are a class of compounds that had numerous commercial uses in the U.S. from 1929 until their prohibition in 1979 [1,2]. Although their most common application was as an insulating fluid in transformers, capacitors, and other electric equipment, PCBs were also used as a plasticizer in open systems that included numerous building materials. Over 70 million kilograms (kg) of PCBs were sold from 1958-1971 for use in adhesives, caulk, ceiling tiles, paint, and sealants [3-6]. PCBs in caulk and other sealants often exceed 1% by weight [7] and migrate from their source products creating the potential for exposure [8].

Approximately 55,000 (46%) public and private schools in the U.S. were constructed during the period when PCBs were sold for open system use [9,10]. Volatilization of PCBs from construction materials has been reported to produce PCB levels in indoor air up to 20 micrograms per cubic meter (μg/m^3^), four orders of magnitude greater than levels typical of ambient air [11-15]. Indoor air PCB concentrations in some schools have been reported to exceed health protective benchmarks suggested by the U.S. Environmental Protection Agency (EPA) [16]. The discovery of PCBs in indoor air of schools can produce numerous complications including mobilization of financial and human resources needed to comply with applicable regulations, disruption of normal operations, and potential risks to health. Identifying effective strategies for managing inhalation exposures associated with PCB-containing building materials is therefore an important public health priority.

The objective of this paper is to report on a longitudinal assessment of PCBs in indoor air of a school building, focusing on (1) the efficacy of selected engineering controls implemented to mitigate concentrations of PCBs in indoor air and (2) the temporal dynamics of PCBs in indoor air. The empirical findings from this assessment have implications for management of PCB-containing materials in schools and other buildings.

## Methodology

### Setting

This intervention was conducted in an elementary school occupied by approximately 455 students in kindergarten through 5^th ^grade and staff. The 6,000 square meter (m^2^) single-story school building contains 24 classrooms and common areas constructed circa 1961 and two masonry and three modular classrooms built after 1978. The envelope of the original structure consists of alternating sections of brick over concrete block and curtain wall. The curtain wall is composed of aluminum framing, single pane glass windows, and mineral fiber (transite) panels. Classrooms, the library, gymnasium, and kitchen are located along the perimeter of the building. Hallways, specialty rooms (e.g., music and art), staff offices and restrooms comprise the remaining interior space.

Unit ventilators with internal fans and steam radiators are used to ventilate and heat the classrooms. The building is not air conditioned. Each classroom has a thermostat that establishes the temperature set point for the ventilation and heating systems. During the heating season, the steam boiler is powered and thermostat set points are set to 20 degrees Celsius (°C). At other times, thermostats are set to 17°C. When set points are met, the outdoor air dampers in the unit ventilators supply an approximate 50:50 mix of recirculated air and outdoor air to classrooms. When room temperatures exceed thermostat set points, outdoor air dampers open fully and supply air is up to 90% outdoor air. Central exhaust systems operate continuously on days when school is in session and remove an average of 5.1 cubic meters of air per minute (m^3^/min) from each classroom.

Transite panels in the curtain wall are set in PCB-containing caulk that fills the channels of aluminum framing. Bulk samples of the caulk were analyzed by EPA Method 8082 and found to contain 1,830 - 29,400 parts per million (ppm) of total PCBs, primarily as Aroclor 1260. The number of transite panels in each classroom ranged from 3 to 9, depending upon the size and configuration of the room. With each transite panel having an area of 0.8 m^2^, there was 10 to 30 linear meters of caulk on the interior face of the exterior wall in each room. Beads of caulk were approximately 5 millimeters (mm) wide, totaling 0.05 to 0.16 m^2 ^of PCB caulk on the interior of each room. Fluorescent light ballasts in the building did not contain PCBs. PCBs were found in ceiling tiles, plastic cove base, and floor tile mastic as well, but at concentrations less than 150 ppm and generally two orders of magnitude lower than the levels present in caulk.

### Design

Three interventions intended to mitigate concentrations of PCBs in indoor air were evaluated in series: ventilation; contact encapsulation; and physical barriers. Indoor air samples from multiple treatment and control classrooms were collected before and after each intervention and analyzed for PCBs. Prior to the first intervention, air sampling was conducted on August 25 - 27, 2010 during which air exchange rates in individual classrooms ranged from 0.4 to 5.9 per hour (h^-1^) and the sampling period-average ambient temperature ranged between 18 - 25°C.

#### Ventilation

**Figure 1:**
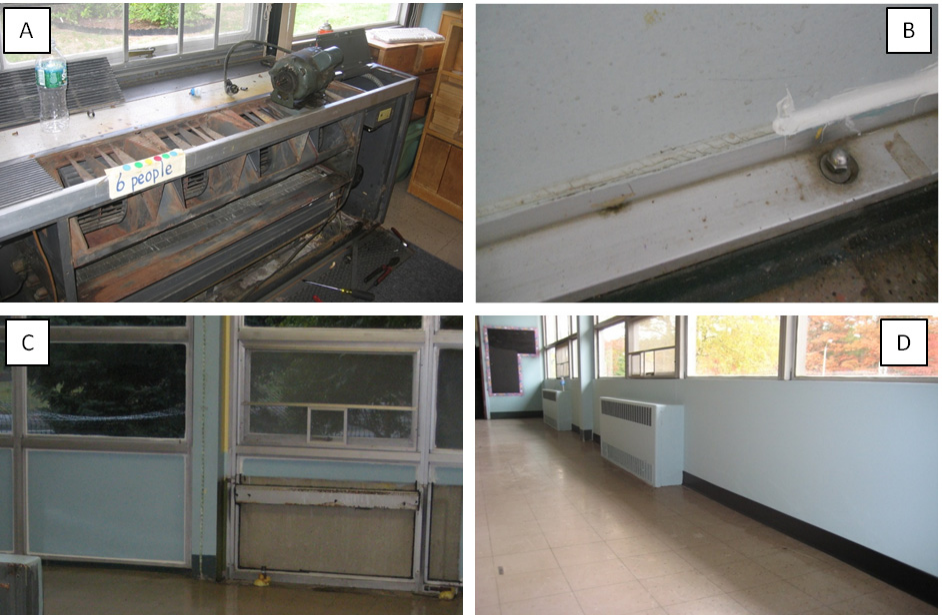
**Photographs of interventions: A) unit ventilator with cover and fan motor removed; B) detail of PCB caulk in original and encapsulated form; C) curtain wall with PCB caulk directly accessible to room (left side) and located behind a convective heater that was removed (right side); (D) finished false wall constructed over encapsulated PCB caulk, transite panels, and aluminum framing**.

Following the pre-intervention sampling, ventilation rates in classrooms were increased by replacing filters, repairing fans in the unit ventilators (Figure 1, Panel A), and adding supplemental ventilation with high efficiency particulate air (HEPA)-filtered outdoor air in one room. Post-intervention sampling for ventilation was conducted on September 6, 2010. During the sampling, air exchange rates (AER) in classrooms ranged from 2.7 to 12 h^-1 ^and ambient temperature was 24°C.

#### Contact encapsulation

After adjusting ventilation rates to within design levels in classrooms throughout the school, we evaluated whether encapsulating PCB caulk would influence PCB levels in indoor air. For this objective, interior-facing beads of caulk were sealed with adhesive-backed polyethylene tape which in turn was covered with a bead of silicone caulk (Figure 1, Panel B). Encapsulation was completed and evaluated in two stages. First, PCB caulk directly accessible to classrooms (approximately 75% of interior caulk in each classroom) was encapsulated (left side of Panel C, Figure 1). Afterwards, indoor air samples were collected on September 19, 2011 when the ambient temperature was 21°C. Next, PCB caulk located behind unit ventilators and convective heaters was encapsulated (right side of Panel C, Figure 1) and indoor air samples were collected on September 29, 2011 at an ambient temperature of 25°C.

#### Physical barrier

For the third intervention, a false wall was constructed over the encapsulated caulk (Figure 1, Panel D). In addition to the potential to reduce indoor air PCB concentrations further, the intent of the false wall was also to limit direct contact with and disturbance of encapsulated PCB caulk. To construct a false wall, aluminum-backed fiberglass insulation board was inserted over the interior face of a transite panel and sealed with silicone caulk. Gypsum board was affixed over the insulation board and the surrounding aluminum frame. The gypsum board was sealed with silicone caulk and then coated with latex paint. False walls were constructed in classrooms during November 2010. Post-intervention sampling was conducted after construction of the false walls was completed. Indoor air samples were collected on November 4, 11, 20, and 24, 2010, and December 2, 2010. Corresponding ambient temperatures were 9.3, 8.7, 9.7, 6.7, and 1.7°C, respectively.

#### Longitudinal assessment

To evaluate whether the mitigation measures were effective over time, we conducted longitudinal follow-up sampling in the building. Nine indoor sampling events were carried out from February 23, 2011 through December 29, 2011; ambient temperatures ranged from -2.1 to 28°C. Three to ten classrooms were sampled during each event.

### Data collection

Ninety-six (96) samples of school-day average PCBs in indoor air of the elementary school were collected during 18 sampling events from August 25, 2010 - December 29, 2012. The samples were collected in accordance with EPA Method TO-10A. School-day in this context corresponds to 6.5 hour sampling durations that typically began between 9:00 a.m. and 10:00 a.m. An outdoor air sample was collected on each day of indoor air sampling. Sampling pumps and cartridges were suspended from aluminum tripods 1 meter above floor level. Pump flows were set for 4 liters per minute and verified by a calibrated flow meter [Bios Drycal] at the start and end of the sampling period. The beginning and ending flow rates were averaged to calculate the total volume of air sampled. Samples were assayed by gas chromatography-mass spectrometry for PCB homologs following EPA Method 8270 C-SIM [Alpha Analytical, Mansfield, MA]. Results were reported in nanograms per cubic meter (ng/m^3^) for each homolog and for total PCBs as the sum of the individual homologs. Homologs reported by the laboratory as non-detect were treated as zero when computing total PCB concentrations in air.

Windows and doors were closed and unit ventilators were operated at a fixed fan speed during all sampling events. Outdoor air delivery rates through the unit ventilator in each room was measured with a calibrated balometer and used to estimate AER. The accuracy of the AER estimates was confirmed by tracer gas following the American Society for Testing and Materials (ASTM) Standard E741-00, *Standard Test Method for Determining Air Change Rate in a Single Zone by Means of a Tracer Gas Dilution Method *conducted in a subset of the classrooms. The average ambient temperature during each sampling event was obtained from an Automated Surface Observing Station (ASOS) weather station located 4 km west of the school.

### Data analysis

Indoor air samples from multiple classrooms were collected before and after each intervention and analyzed for total PCBs. All total PCB concentrations were greater than the limit of detection. To ensure comparability of data among monitoring periods, the analysis was based on rooms in the original building with operating unit ventilators and transite panels set in aluminum framing with PCB caulk.

Ventilation rate and temperature have been reported elsewhere to have an influence on PCB levels in outdoor and indoor air [17-19]. To account for this effect when evaluating efficacy of interventions, measured concentrations were normalized from the measured AER to an AER of 11.3 m^3^/min and for vapor pressure from indoor temperature to a temperature of 20°C. On sampling days that ambient temperature exceeded 20°C (the thermostat set point) indoor temperature was assumed to be equal to ambient temperature. On other days, indoor temperature was assumed to be 20°C. PCB vapor pressure was estimated with the Clausius-Clapeyron equation from temperature-specific vapor pressures compiled by Li [20] and a homolog mixture for Aroclor 1260 reported by ATSDR [21]. The heat of evaporation estimated for Aroclor 1260 was 82 kilojoule per mole (kJ/mol).

Pre- and post-intervention measurements were not always made in the same room. Therefore, two analyses were used to evaluate pre-intervention and post-intervention concentrations of PCBs in indoor air. In one analysis, data from all rooms sampled pre- and post-intervention were used to test the null hypothesis that median concentrations of PCBs in indoor air were equal before and after an intervention (Wilcoxon Rank Sum test). In the second analysis, paired pre- and post-intervention measurements were analyzed using the sign test which allowed for control of room-specific factors that might influence PCB concentrations (n = 3-8 pairs depending on the intervention).

The analysis of variability over time was performed by first compiling summary statistics for airborne PCB levels, AER, surface area of PCB caulk in a classroom, and ambient temperature. A linear mixed effects model controlling for repeated measures from individual classrooms was used to test for significant variability of indoor air PCB concentration with ambient temperature, AER, and surface area of caulk per classroom. The relationship between ambient temperature and PCB levels in indoor air was evaluated through additional analysis with regression models.

### Quality assurance

To ensure traceability and accuracy of the data, a series of quality assurance steps was performed. A chain of custody (COC) form followed each air sample from the field, to the laboratory, and finally to the database manager. Sample collection and analysis procedures were performed in accordance with quality assurance measures prescribed by EPA Method TO-10A. The DL ranged from 5-10 ng per homolog (nominally 10 ng/m^3 ^in equivalent air concentration) over the course of investigation. Recovery efficiency was measured using fortified samples. Two surrogates, Cl3-BZ#19-C13 and Cl8-BZ#202-C13, were added to each sample and their average recoveries were 93% (SD = 22%) and 86% (SD = 18%), respectively (N = 142). PCBs were not detected in any field blank samples (N = 18). The precision of samples was expressed as the root mean square error (RMSE) between paired primary samples and duplicates. Paired samples showed good agreement with a slope of 0.97, RMSE of 36.5 ng/m^3^, and coefficient of variation of 14.6%. An outdoor air sample was collected during each sampling event and total PCB concentrations ranged from 3.8 to 10.4 ng/m^3^. To ensure the encapsulant was effectively blocking the release of PCBs into classrooms, wipe samples were collected on the encapsulated surfaces periodically during the longitudinal study. A total of 70 of these wipe samples were analyzed by the EPA Method 8082 and all of the samples were below 1 microgram per 100 square centimeters (μg/100 cm^2^), which was the detection limit.

## Results

### Efficacy of interventions

**Figure 2:**
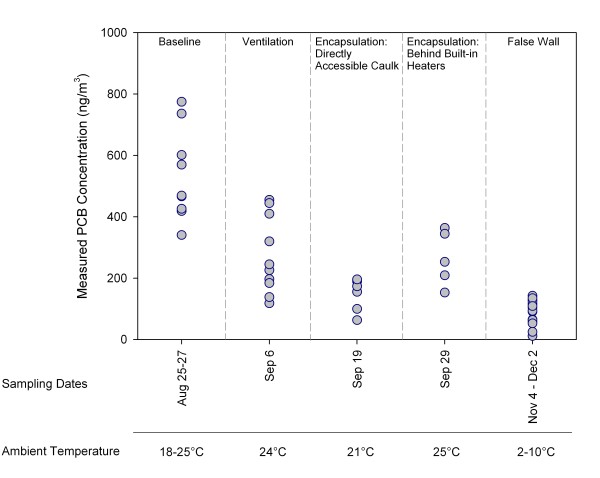
**Measured concentrations of total PCBs in indoor air of classrooms at baseline and following implementation of engineering controls**.

The efficacy of ventilation and encapsulation was evaluated over the first 8 of the 18 total sampling events conducted at the school. Measured concentrations for the individual samples collected across the eight sampling events from August 25 - December 2, 2010 are plotted in Figure 2. At baseline, the average PCB level in indoor air was 533 ng/m^3 ^with a coefficient of variation (CV) of 28%. Concentrations were lower on September 6, 2010 following improvements to ventilation throughout the school (mean 274 ng/m^3^, CV 46%) and again on September 19, 2010 after encapsulation of PCB caulk that was directly accessible in classrooms (mean 153 ng/m^3^, CV 34%). Average airborne PCB levels were 265 ng/m^3 ^(CV 34%) in the samples collected on September 29, 2010 after encapsulating caulk located behind heaters, unit ventilators, and built-in cabinets. The average airborne PCB level was 95 ng/m^3 ^(CV 40%) following construction of the false walls.

Median normalized concentrations and a comparison of pre- and post-intervention results according to the Wilcoxon Rank Sum test are shown in Table 1. Improvement in ventilation rates was associated with a statistically significant reduction (66%, 432 to 149 ng/m^3^, p < 0.01) in the normalized median concentration of PCBs in indoor air. Encapsulation of directly accessible interior caulk yielded a marginally significant (p = 0.08) reduction in the normalized median indoor air PCB concentration (22%, 282 to 220 ng/m^3^). Encapsulation of interior caulk behind convective heaters, unit ventilators, and built-in furniture reduced normalized indoor air concentrations by an additional 40% (220 to 133 ng/m^3^, p = 0.02). The normalized median concentration of airborne PCBs in classrooms was 76 ng/m^3 ^following construction of false walls and was significantly different from full encapsulation of caulk.

**Table 1:** Median concentration of polychlorinated biphenyls in indoor air before and after implementation of three mitigation measures, normalizing for temperature and ventilation rates.

Period	Number of Rooms	Median (ng/m^3^)^1^	H_0_: medians are equal (p-value)^6^
**Ventilation^2^**			
August 25-27, 2010	9	432	< 0.01
September 6, 2010	10	149	
**Encapsulation: Directly Accessible Caulk^3^**			
September 6, 2010	10	282	0.08
September 19, 2010	7	220	
**Encapsulation: Behind Built-in Equipment and Furniture^4^**			
September 19, 2010	7	220	0.02
September 29, 2010	5	133	
**False Wall^5^**			
September 29, 2010	5	133	< 0.01
Nov. 4 - Dec. 2, 2010	19	76	

^1 ^Median of temperature-normalized and ventilation-normalized concentrations

^2 ^Ventilation rates were improved by replacing filters and repairing fans in the unit ventilators. Median of temperature-normalized, but not ventilation-normalized concentrations.

^3 ^PCB-containing caulk along the interior face of transite panels open to classrooms was covered with adhesive-backed polyethylene tape and sealed with silicone caulk.

^4 ^PCB-containing caulk located behind unit ventilators and convective heaters was covered with adhesive-backed polyethylene tape and sealed with silicone caulk.

^5 ^False walls constructed over encapsulated PCB caulk and adjoining transite panels and framing.

^6 ^Wilcoxon rank sum test of the hypothesis that the median pre-intervention and post- intervention concentrations are equal.

**Figure 3:**
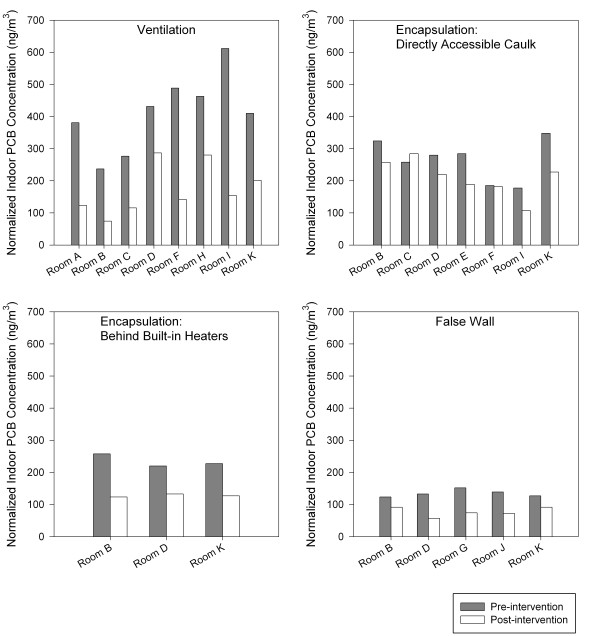
**Measured concentrations of total PCBs in indoor air of paired rooms before and after increased ventilation; encapsulation of PCB caulk exposed to classrooms; encapsulation of PCB caulk behind unit ventilators, convective heaters, and built-in shelves; and construction of false walls**.

Similar results were obtained when the analysis was restricted to matched pairs of rooms from the pre- and post-intervention datasets (sign test). Room-specific pre- and post- intervention concentrations of PCBs in indoor air are shown in Figure 3. Increased ventilation (pre- and post-means of 423 ng/m^3 ^and 173 ng/m^3^) and partial encapsulation (pre- and post- means of 265 ng/m^3 ^and 210 ng/m^3^) were associated with statistically significant (p ≤ 0.01) reductions in airborne levels of PCBs. The reductions associated with full encapsulation (pre- and post-means of 251 ng/m^3 ^and 169 ng/m^3^, p = 0.25) and false wall construction (pre- and post-means of 135 ng/m^3 ^and 77 ng/m^3^, p = 0.06) were not statistically significant; however, only small numbers of paired rooms (3 and 5, respectively) were available for these interventions, which limited the statistical power of the hypothesis tests.

### Longitudinal assessment

Summary statistics for measured PCB concentrations in indoor air of classrooms, AER, and ambient temperature over a 14-month period following adjustment of ventilation, encapsulation of PCB caulk, and construction of false walls are shown by date of sampling in Table 2. Average PCB concentrations were approximately 100 ng/m^3 ^from November 2010 through April 2011, 167 to 276 ng/m^3 ^from May 21 - July 13, 2011, and approximately 100 ng/m^3 ^through the remaining sampling events in 2011. The highest average PCB concentrations in indoor air were observed when outdoor temperatures were above 20°C. Air exchange rates averaged 3.3 h^-1 ^when ambient temperature was greater than 20°C and 2.9 h^-1 ^at lower temperatures.

**Table 2:** Ambient temperature and summary statistics for PCB concentrations (ng/m^3^) in indoor air of classrooms of an elementary school over a 14-month period

Sample Date	Temperature (Celsius)	AER (h^-1^)	N	Mean	Standard Deviation	Coefficient Of Variation	Min	Max
11/4/2010	9.3	2.0	1	105	--	--	105	105
11/11/2010	8.7	2.8	7	83	40.5	48.8	12	128
11/20/2010	9.7	2.4	8	103	40.6	39.7	25	142
11/24/2010	6.7	2.9	2	94	58.0	61.9	53	135
12/2/2010	1.7	4.3	1	109	--	--	109	109
2/23/2011	1.8	3.1	5	101	27.2	26.9	76	146
4/20/2011	6.0	2.4	3	109	23.9	22.0	93	136
4/21/2011	11	2.0	3	84	46.6	55.5	44	135
5/21/2011	22	3.6	8	167	44.8	26.8	103	224
6/9/2011	27	3.1	3	276	117.5	42.6	152	386
7/13/2011	28	3.6	3	275	87.3	31.8	175	337
7/14/2011	22	2.9	3	99	69.7	70.3	43	177
10/7/2011	14	3.8	8	79	21.0	26.6	52	114
12/29/2011	-2.1	2.8	10	56	20.9	37.5	11	85

N = number of observations; Min = minimum concentration; Max = maximum concentration

In a linear mixed effects model controlling for repeated measures from classrooms, natural log PCB concentration was significantly associated with ambient temperature (p < 0.001) and not significantly associated with AER (p = 0.16) or the ratio of caulk surface area to room volume (p = 0.29). Concentrations within a room were not correlated over time (intra-class correlation coefficient of -0.15). Therefore, subsequent analyses focused on variability of sampling event average concentrations rather than results from individual classrooms.

**Figure 4:**
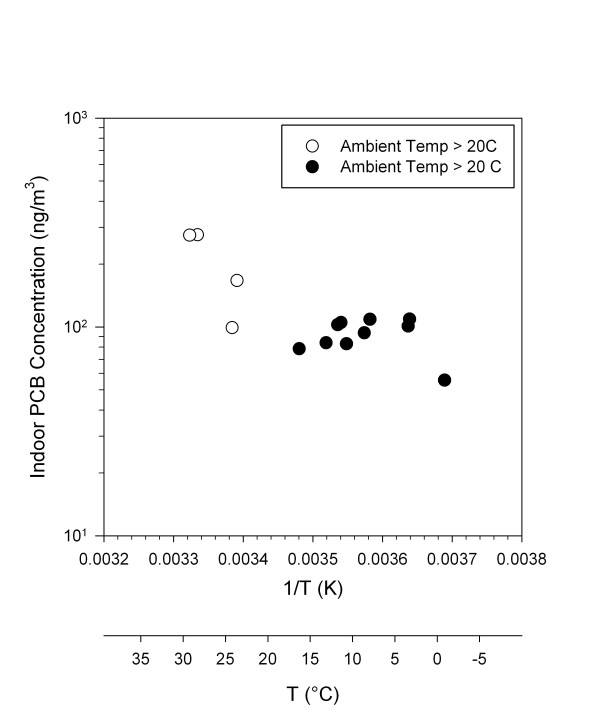
**Average PCB concentration plotted against the inverse of ambient temperature (K)**.

After the interventions were completed, the average PCB level in indoor air by sampling event ranged from 79 to 276 ng/m^3 ^and varied significantly (p < 0.05) over time (linear mixed effects model). Ventilation-normalized PCB concentrations in indoor air of classrooms in the school were directly related to ambient temperature as shown in Figure 3. Examination of the plot suggests that indoor air PCB concentrations reflect two distinct conditions. Natural log PCB levels in indoor air varied approximately linearly with ambient temperature above 20°C (open symbols in Figure 4), but have little association with ambient temperatures below 20°C (filled symbols in Figure 4). A "broken-stick" regression model with a node at 20°C explained 79% of the variability of the indoor PCB concentration (p < 0.001). The slope was -6.2 × 10^3 ^natural log ng/m^3 ^per 1/K (Kelvin) for temperatures above 20°C and -6.3 × 10^2 ^natural log ng/m^3 ^per 1/K for temperatures below 20°C. The higher temperature regime corresponds to periods when indoor air temperatures are approximately equal to ambient temperature (because the building is not air conditioned), while the lower temperature range corresponds to periods when indoor air temperatures are maintained at approximately 20°C by the central heating system.

## Discussion

In this 16-month long investigation, we assessed the efficacy of engineering controls employed to reduce concentrations of airborne PCBs inside of a school. We found that improving ventilation with outdoor air and encapsulating PCB-containing caulk located on the inside face of the building envelope yielded reductions of PCBs levels in indoor air. Implementation of these engineering controls throughout the school also led to a reduction in the variability of indoor air PCB concentrations in classrooms. After adjusting ventilation and erecting physical barriers over the interior-facing PCB caulk, indoor air PCB levels were found to be directly related to temperature.

The mitigation methods employed in this school building reduced indoor air PCB concentrations by approximately 87% from initial concentrations of 299 - 1,800 ng/m^3^. This reduction was meaningful as concentrations were brought below health protective guidelines for schools suggested by EPA [16]. Moreover, the resulting concentrations were below the annual average guideline value of 230 ng/m^3^, that was derived through a site-specific risk assessment for this school. The initial concentrations in the school were similar to airborne indoor PCB concentrations reported for other buildings constructed with PCB-containing caulk, e.g., 120 - 320 ng/m^3 ^in a laboratory [11], 715 - 2253 ng/m^3 ^in unidentified types of buildings [22], < 100 - 3,000 ng/m^3 ^for public buildings in Switzerland [18], 11 - 393 ng/m^3 ^in a university building [8] and 78 - 1406 ng/m^3 ^in New York City schools [23]. Consequently, the mitigation methods found to be effective here may also be efficacious elsewhere.

Minimal disruption of operations is an important objective of maintenance, construction, and remediation activities in buildings. The modification of ventilation systems and encapsulation of source materials described in this paper were conducted outside of school hours and therefore did not interfere with regular school activities. Common remediation methods for PCBs in buildings materials involve removal or chemical treatment of PCB caulk and surrounding materials such as brick, concrete, and mortar [24]. Noise, dust, vibration, chemicals, and mechanical removal of building components associated with these abatement methods often require buildings to be evacuated for weeks or months. Our findings demonstrate that risks of PCBs can be managed without significant disruption of building operations. The benefits of mitigation of PCB-containing materials rather than abatement have been noted previously [25]. Less disruption is expected to translate to lower costs of remediation although to our knowledge a rigorous comparison has not yet been made for costs of mitigation and abatement of PCBs in building materials.

After improving ventilation and encapsulating interior PCB-containing caulk throughout the school, concentrations of PCBs in indoor air did not vary significantly across rooms in the building but were found to vary significantly over time. Subsequent analyses demonstrated that variation in ambient temperature explained 79% of the temporal variability of school-wide average indoor air concentrations of PCBs. An association between PCB levels in indoor air and temperature has been reported previously. In one room of a prefabricated concrete office building sealed with PCB-containing caulk, indoor air PCB levels were reported to be associated with ambient temperature [12]. Likewise, Kohler et al. [18] found a 'slight trend' between indoor airborne PCB levels and room temperature, although the analysis could not account for other factors such as air exchange rate. Seasonal differences in indoor air concentrations of PCBs have been ascribed to temperature as well [7,8].

In our investigation, PCB concentrations (natural log ng/m^3^) in indoor air decreased by 6.2 x10^3 ^for every 1/K decrease in ambient temperature above 20°C. According to the Clausius-Clapeyron equation and our estimated heat of evaporation of 82 kJ/mol for Aroclor 1260, natural log PCB vapor pressure would increase by -9.8 × 10^3 ^per 1/K decrease in temperature. The difference between the theoretical and observed slopes may result from differences between ambient temperature and the temperature of materials inside the building to which PCBs are adsorbed. When ambient temperatures were below 20°C and classroom temperatures were maintained by convective heating, PCB levels in indoor air were effectively constant. This observation indicates that airborne PCB concentrations in the building are only indirectly related to ambient temperature and instead directly a function of temperature in the indoor environment.

Additional investigation of residual sources of PCBs, including PCBs absorbed into other building materials over time and the effect of temperature on flux or concentration, may be useful in understanding ongoing PCB indoor air contamination following remediation. In this school, the continued presence of indoor air PCB levels above corresponding concentrations in outdoor air indicate that residual sources of PCB emissions remain within the building. Identification of ongoing PCB releases in the school was outside the scope of the present investigation. However, screening for potential source materials conducted at the outset of the investigation revealed PCBs at concentrations below 50 parts per million in bulk samples of ceiling tiles and mastic below floor tiles. These materials could be contributing to the residual PCBs observed in indoor air of the school.

The empirical findings from this longitudinal assessment have significant implications for management of PCB-containing building materials. First, relatively low cost engineering controls were found to reduce indoor air concentrations of PCBs substantially. Moreover, these mitigation methods were shown to be effective for at least 1-year. Management in-place methods such as these avoid the disruption and higher costs of demolition, disposal, and reconstruction required when PCB-containing building materials are removed from a school. Second, we found that short-term measurements, such as over a single day or season, may not be representative of long-term average or typical conditions in a building because ventilation and/or ambient temperature during sampling can have a strong influence on PCB levels in indoor air. The reliability of short-term measures to represent long-term average exposure is important in this situation because health risks of PCBs are considered to be a function of chronic exposure [26]. Air sampling conducted when ventilation systems are not fully operational or when temperatures are elevated may lead to overestimates of risk. Conversely, air sampling conducted when ventilation rates are atypically high or temperatures are atypically low could lead to an incorrect determination that no actions are indicated based upon a measurement from a single point in time. In either case, risk assessment results based on short-term measurements may be erroneous and could lead to sub-optimal allocation of resources. To assess long-term average exposure more accurately, the ventilation system should be operated during testing in a manner that it is intended to operate in the future. Likewise, temperatures at the time of sampling should be similar to school-year average temperatures.

## Conclusions

We evaluated the efficacy of selected engineering controls intended to mitigate concentrations of PCBs in indoor air of a school building. Concentrations of PCBs in indoor air were reduced by increasing outdoor air ventilation, encapsulating PCB-containing caulk located on the inside face of the building envelope, and physical barriers constructed over encapsulated caulk. The combination of ventilation, encapsulation, and physical barriers controlled indoor air levels of PCBs effectively over the course of this 16-month investigation. Indoor air PCB levels were strongly associated with the temperature of ambient and indoor air following implementation of the mitigation measures. The results of this assessment have significant implications for the assessment and management of PCBs in building materials.

## Competing interests

The authors declare that they have no competing interests.

## Authors' contributions

DM conceived of the study, led the analysis, and drafted the manuscript. TM participated in the design of the study, performed the statistical analysis, and drafted portions of the manuscript. MF participated in the design of the study and collected the data. JA collected a portion of the data and contributed to drafting the manuscript. KM participated in the design of the study. JS contributed to the design and statistical analysis. JM participated in the design of the study. All authors read and approved the final manuscript.
